# Allergic bronchopulmonary mycosis induced by pembrolizumab for bladder cancer: A case report

**DOI:** 10.1016/j.rmcr.2025.102179

**Published:** 2025-02-10

**Authors:** Hiroki Iida, Kenichiro Atsumi, Naohiro Kadoma, Sae Takashima, Yukari Shirakura, Ayana Suzuki, Kakeru Hisakane, Ryo Matsuoka, Koji Nagata, Masahiro Seike, Takashi Hirose

**Affiliations:** a Department of Pulmonary Medicine and Medical Oncology, Nippon Medical School Tama Nagayama Hospital, 1-7-1 Nagayama, Tama-shi, Tokyo, 206-8512, Japan; b Department of Urology, Nippon Medical School Tama Nagayama Hospital, 1-7-1 Nagayama, Tama-shi, Tokyo, 206-8512, Japan; c Department of Pathology, Nippon Medical School Tama Nagayama Hospital, 1-7-1 Nagayama, Tama-shi, Tokyo, 206-8512, Japan; d Department of Pulmonary Medicine and Oncology, Graduate School of Medicine, Nippon Medical School, 1-1-5 Sendagi, Bunkyo-ku, Tokyo, 113-8603, Japan

**Keywords:** Aspergillosis, Allergic bronchopulmonary, Pembrolizumab, Urinary bladder neoplasms, Immune checkpoint inhibitors, Drug-related side effects and adverse reactions, Programmed cell death 1 receptor

## Abstract

Pembrolizumab is an immune checkpoint inhibitor (ICI) of programmed cell death-1 category, used for treating various types of cancer. ICIs can sometimes result in immune-related adverse events. Allergic bronchopulmonary mycosis (ABPM) induced by ICI has rarely been reported. We hereby report the case of an 83-year-old woman who experienced non-Aspergillus ABPM with eosinophilia induced by pembrolizumab that had been prescribed for treating bladder cancer. Steroid monotherapy with prednisone was successful and pembrolizumab could be resumed. Through the present case report, we urge the clinicians to be aware of the potential risk of ABPM as a T-helper type 2-associated immune-related adverse event.

## Highlights

• We hereby report a case of non-Aspergillus ABPM induced by pembrolizumab.
• Steroid monotherapy was successful and pembrolizumab could be resumed.
• Clinicians should be aware of the potential risk of ABPM as a Th2-associated irAE.

## 1 Introduction

Pembrolizumab is an immune checkpoint inhibitor (ICI) that binds to the programmed cell death-1 (PD-1) protein and is used to treat various cancers. Treatment with ICIs can result in autoimmune phenomena known as immune-related adverse events (irAEs) [1]. The frequency of eosinophil-mediated organ damage in an irAE is relatively low. Allergic bronchopulmonary mycosis (ABPM) is a chronic airway disease characterized by the colonization of the airways by fungal species, and induces type I and III hypersensitivity reactions. ABPM induced by ICI has rarely been reported. We hereby report the case of a patient suffering from non-Aspergillus ABPM induced by pembrolizumab that had been prescribed for bladder cancer.

## 2 Case presentation

An 83-year-old Japanese woman with no previous history of asthma or smoking presented to our hospital with microscopic hematuria. Histological examination by a transurethral resection of the bladder tumor (TUR-BT) revealed a high grade (G2 > G3, pT2, Ly0, and V0) invasive urothelial carcinoma with glandular differentiation. Whole-body computed tomography (CT) revealed a diagnosis of muscle invasive bladder cancer pT2N0M0 (stage II). Due to her age and performance status, the patient opted for palliative combination therapy including re-TUR-BT, gemcitabine/cisplatin combination chemotherapy, and concurrent radiation therapy (33 Gy in 10 fractions). After the completion of one cycle of chemotherapy, she was diagnosed with progressive disease and received intravenous pembrolizumab 200 mg every three weeks as a second-line therapy. She had mild eosinophilia (100–400/μL) at diagnosis of bladder cancer, which progressed to intermittent eosinophilia (400–800/μL) following the initiation of pembrolizumab therapy.

**Fig. 1:**
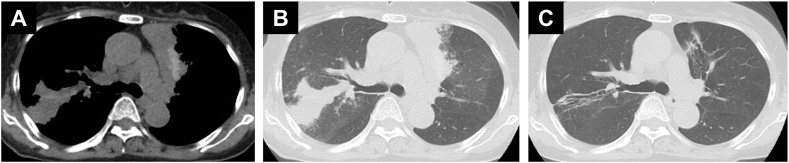
(A, B) Chest computed tomography at the first examination revealing an infiltration with high-attenuation mucus plugs in the bilateral upper lobes. (C) After nine weeks of steroid treatment, chest computed tomography revealing no evidence of infiltration and mucus plugs.

**Fig. 2:**
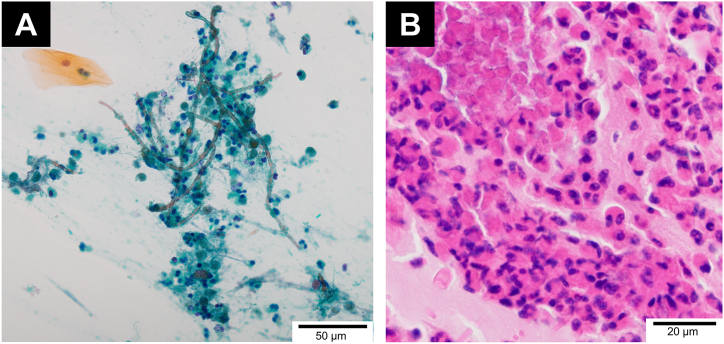
(A) Cytology of the bronchial lavage fluid indicating the presence of branching hyphae. (B) Hematoxylin-and-eosin (H&E) staining of the specimens obtained by transbronchial lung biopsy revealing an inflammatory cell infiltrate consisting mainly of lymphocytes and plasma cells in the alveolar regions and an eosinophilic infiltrate in the bronchiolar regions and mucus.

After the 25th cycle of pembrolizumab therapy, she suffered from a wet cough, slight wheezes, and was febrile with a temperature of 38 °C. Chest X-ray indicated bilateral consolidation, and chest CT revealed infiltration with high-attenuation mucus plugs in the bilateral upper lobes (Fig. 1A and B). The laboratory data (Table 1) revealed a peripheral blood eosinophil count of 1850/μL. Her total IgE levels were not elevated (110 IU/mL) and her test result for the specific IgE against Aspergillus was negative. She had elevated fraction exhaled nitric oxide levels (45 ppb). Bronchoscopy revealed mucus plugs in the bilateral upper bronchi. Bronchoalveolar lavage (BAL) was performed in the right upper lobe, and the BAL fluid revealed elevated eosinophil ratio (15 %). The cytology of the BAL fluid indicated branching hyphae (Fig. 2A). A transbronchial lung biopsy was randomly performed from the right upper lobe and the reports revealed an inflammatory cell infiltrate consisting mainly of lymphocytes and plasma cells in the alveolar regions and an eosinophilic infiltrate in the bronchiolar regions along with mucus (Fig. 2B). No fungal species was identified in the sputum cultures.

**Table 1:** Laboratory data.

**Hematology**		**Serology**	
WBC	7400/μL	KL-6	345 U/mL
Neutrophils	46.0 %	SP-D	70.2 ng/mL
Lymphocytes	11.0 %	IGRA	(−)
Eosinophils	25.0 %	Anti-MAC antibody	<0.5 U/ml
Monocytes	17.0 %	HIV	(−)
Basophils	1.0 %	β-D-glucan	4.8 pg/mL
RBC	328 × 10^4^/μL	*Aspergillus* antigen	0.3
Hb	9.8 g/dL	*Aspergillus* IgG antibody	(−)
Plt	32.2 × 10^4^/μL	IgE	110 IU/dL
		Specific IgE	
**Biochemistry**		*Penicillium*	Class 1
Alb	3.1 g/dL	*Aspergillus*	Class 0
AST	29 U/L	*Cladosporium*	Class 0
ALT	16 U/L	*Mucor*	Class 0
LDH	223 U/L	*Alternaria*	Class 0
CRP	7.41 mg/dL	*Trichophyton*	Class 0
BUN	13.4 mg/dL		
Cre	0.57 mg/dL		
HbA1c	5.6 %		

Abbreviations: KL-6, Krebs von den Lungen-6; SP-D, surfactant protein D; IGRA, interferon-gamma release assays; MAC, mycobacterium avium complex; HIV, human immunodeficiency virus.

**Fig. 3:**
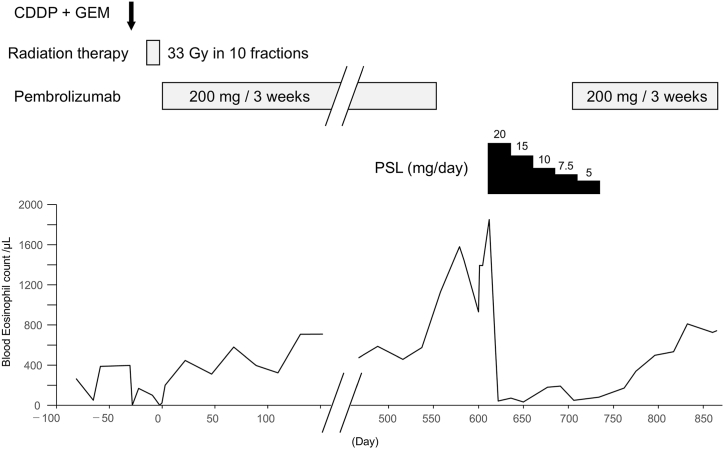
Clinical course. After four months of steroid therapy, the peripheral blood eosinophil count lowered. The patient is currently continuing pembrolizumab therapy. Abbreviations: CDDP, cisplatin; GEM, gemcitabine; PSL, prednisone.

A diagnosis of ABPM was made based on the satisfaction of the following six of the 10 diagnostic criteria proposed by Asano et al. [2]: (1) presence of asthmatic symptoms; (2) peripheral blood eosinophilia (>500/μL); (3) filamentous fungal growth in BAL fluid; (4) presence of fungal hyphae in bronchial mucus plugs; (5) presence of mucus plugs in the central bronchi revealed by CT/bronchoscopy; and (6) high-attenuation mucus in the bronchi on CT. She was prescribed 20 mg of prednisone in the form of once-daily oral treatment. The dose of prednisone was reduced by 2.5–5 mg, with continuous monitoring of the imaging and blood test data (Fig. 3). After nine weeks of treatment with a dose of 5 mg of prednisone, chest CT indicated an absence of infiltration and mucus plugs (Fig. 1C) and the peripheral blood eosinophil count also reduced. She was able to resume intravenous pembrolizumab 200 mg every three weeks. The following month, steroid therapy was discontinued. The patient is currently continuing pembrolizumab therapy and has not experienced any recurrence of lung lesions.

## 3 Discussion

In this case study, we have reported a case of ABPM with eosinophilia induced by pembrolizumab that was prescribed for treating bladder cancer in an 83-year-old woman. The steroid monotherapy with prednisone was successful and pembrolizumab could be resumed. To the best of our knowledge, this is the first case report to report a case of non-Aspergillus ABPM induced by ICI. Pembrolizumab is a PD-1 antibody that inhibits the binding of PD-1 to programmed cell death ligands 1 (PD-L1) and PD-L2. In cancer cells, the expression of PD-L1 sends inhibitory signals that suppress T-cell activation through PD-1, thereby enabling the tumor cells to evade the immune system. Recent studies have demonstrated that T-helper type 2 (Th2) inflammation can be facilitated by blocking PD‐1–PD‐L2 interaction using anti‐PD‐1 antibodies [3]. Th2 cells mainly produce interleukins (IL)-4, IL-5, and IL-13, which promote eosinophil recruitment and contribute to the development of Th2-associated lung disease. Thus, anti‐PD‐1 antibodies might possibly mediate Th2‐associated pulmonary diseases. Acute eosinophilic pneumonia triggered by the anti-PD-1 has been reported [4]. ABPM is currently believed to be caused by a hypersensitivity reaction to a fungal species colonizing airways driven by an elevated antigen-specific Th2 response [5]. Two other cases of ABPM induced by ICIs have previously been reported. In the first case, the patient was a 58-year-old man diagnosed with renal cell carcinoma who was treated with an anti-PD-1 antibody therapy (no drug description) [6]. He had no previous history of asthma. His sputum culture tested positive for *Aspergillus fumigatus*, and he was treated with itraconazole and corticosteroids. In the second case, the patient was a 63-year-old man who had been diagnosed with squamous cell lung carcinoma and was treated with pembrolizumab [7]. He had a history of childhood asthma. His sputum culture tested positive for *Aspergillus fumigatus*, and he was treated with voriconazole and corticosteroids.

In the present case, the presence of mycelium was proven, but the fungal species could not be identified. The possibility of a fungal species other than *Aspergillus* species colonizing the airways was considered. ABPM being caused by *Schizophyllum commune* (*S. commune*) was reported in 1994 [8]. A nationwide survey of ABPM in Japan had reported that besides the *Aspergillus species, S. commune* was the most common fungal species [9]. *S. commune* is difficult to identify morphologically, and the diagnosis is often missed because of the featureless colonies in culture. In a review of 143 global cases of non-Aspergillus ABPM, the commonest etiologic agent was identified to be *Candida albicans*, which was reported in 60 % of the cases, followed by *Bipolaris species* (13 %) and *S. commune* (11 %) [10].

The total IgE levels were not elevated in the present case, whereas they were elevated in the previous two cases at 2514 and 7050 IU/dL, respectively [6,7]. A previous study had reported that patients with disease onset at advanced ages had lower total IgE levels [11]. We hypothesized that the current patient's age of 83 years played a role in the low total IgE levels.

The standard treatment of ABPM typically comprises systemic steroid therapy with or without adjunct antifungal agents. In the present case, the fungal species was not identified, hence antifungal agents were not prescribed, and steroid monotherapy was administered. Pembrolizumab could be resumed during steroid therapy. During steroid therapy, the peripheral blood eosinophil counts were low. After completion of steroid therapy, intermittent eosinophilia was observed again (Table 1). Although there was no recurrence of pulmonary lesions, the possibility of potential recurrence in the future cannot be ruled out.

## 4 Conclusions

We have reported a case of non-Aspergillus ABPM with eosinophilia induced by pembrolizumab that was prescribed for treating bladder cancer. Steroid monotherapy with prednisone was successful and pembrolizumab could be resumed. Through the present case report, we urge the clinicians to be aware of the potential risk of ABPM as a Th2-associated irAE.

## CRediT authorship contribution statement

**Hiroki Iida:** Writing – review & editing, Writing – original draft, Investigation, Formal analysis, Data curation. **Kenichiro Atsumi:** Writing – review & editing, Writing – original draft, Project administration, Investigation, Formal analysis, Data curation, Conceptualization. **Naohiro Kadoma:** Writing – original draft, Project administration, Investigation, Formal analysis, Data curation. **Sae Takashima:** Writing – review & editing, Investigation. **Yukari Shirakura:** Writing – review & editing, Investigation. **Ayana Suzuki:** Writing – review & editing, Investigation. **Kakeru Hisakane:** Writing – review & editing, Investigation. **Ryo Matsuoka:** Writing – review & editing, Writing – original draft, Supervision, Investigation, Data curation. **Koji Nagata:** Writing – review & editing, Visualization, Data curation. **Masahiro Seike:** Writing – review & editing, Supervision. **Takashi Hirose:** Writing – review & editing, Supervision, Project administration.

## Funding

This research did not receive any specific grants from funding agencies in the public, commercial, or not-for-profit sectors.

## Declaration of competing interest

The authors declare the following financial interests/personal relationships which may be considered as potential competing interests:M. Seike has received honoraria for lectures from MSD KK. T. Hirose has received honoraria for lectures from MSD KK. If there are other authors, they declare that they have no known competing financial interests or personal relationships that could have appeared to influence the work reported in this paper.
